# Midland Hospitals on the National Insurance Bill

**Published:** 1911-06-24

**Authors:** 


### SPECIAL INSTITUTIONAL ARTICLE.

### MIDLAND HOSPITALS ON THE NATIONAL INSURANCE BILL.

### A UNITED HOSPITAL POLICY.

A meeting of representatives of the hospitals in the Midland district was held in the Board Room of the General Hospital, Birmingham, on Wednesday last week, at which fifty representatives were present. Mr. Neville Chamberlain, of the General Hospital, Birmingham, was voted to the chair on the proposal of Mr. F. S. Goodwin, seconded by Mr. Albert Cay.

#### Why the Meeting was Called.

Mr. Chamberlain pointed out the object of the meeting, and said that at the General Hospital they had appointed a special committee to consider the effect of the National Insurance Bill on the hospital, and that two representatives, Mr. F. S. Goodwin and Mr. Howard Collins, attended the meeting of the British Hospitals Association in London. The result of that meeting was to appoint a committee which was deputed to represent the voluntary hospitals, and if necessary to interview the Chancellor of the Exchequer on the subject. Mr. Howard Collins, the House Governor of the General Hospital, had been appointed a member of that committee, but he seemed to be the sole representative of the hospitals of the Midlands, of which there were forty. It was felt by the Board of the General Hospital that his opinion and remarks would carry less weight if he represented Birmingham alone than if he could speak in the name of the larger body of the voluntary hospitals of the Midlands, and it was with a view of obtaining their opinions on the Bill and to see if they agreed with the opinions of the Birmingham General Hospital Committee that this meeting had been called. Mr. Chamberlain expressed his disappointment that there was no reference in the Chancellor's speech in Birmingham on Saturday last to the question of voluntary hospitals, and pointed out that the only references made by Mr. Lloyd George were mainly to point out the advantage of State insurance in Germany, but Mr. Chamberlain pointed out that hospitals in Germany which correspond with our great hospitals are not voluntary hospitals, but municipal—supported partly by the municipality and partly by the payments of patients themselves. In his opinion it was a rash experiment to spend so much money as was provided for consumption sanatoria, and at the same time to withhold all help from institutions like voluntary hospitals, of the good work of which there could be no doubt, and which stood so much in need of assistance.

Mr. Howard Collins then gave the result of the figures collected from the hospitals in the Midlands: Forty hospitals in the Midland Counties, that is within fifty miles of Birmingham, were communicated with, of whom thirty-two have supplied the information asked for. A summary of these statements shows that of the ten Birmingham hospitals (including the General Dispensary), the proportion of income which is affected by the Bill as drawn is 47.4 per cent. or a total of £34,478, and of thirty-two of the forty hospitals included in the Midland district 51.86 per cent. or £100,700.

#### The Queen's Hospital Resolutions.

The Chairman then moved the formal resolution, which was seconded by Mr. W. A. Crosbee, Chairman of the Queen's Hospital, who mentioned that the committee of the Queen's Hospital had approved of the following recommendations at their own meeting:

1. That this committee considers that the first duty of those responsible for the National Insurance Bill is to make adequate provision for protecting the interests of the hospitals in view of the serious injury which will certainly accrue through the reduction of subscriptions, and calls upon the local members of Parliament to use their influence in this direction.
2. That the hospitals shall receive payment at a defined rate per day for every insured person entering the hospital, and that cases dealt with under the Workmen's Compensation Act shall also be paid for.
3. The hospitals shall confer with the health committees when necessary, and not with the approved societies.
4. That in the event of a serious decrease in contributions without any definite guarantee of grants from other quarters, the Queen's Hospital should not increase its liabilities, but should only maintain as many beds as the financial position permits.

#### The General Discussion.

Councillor Harris (representing the Skin Hospital) strongly supported, remarking that his hospital, like many of the smaller institutions, depended on what was called the shilling registration fee for a large part of their revenue.

Further speeches were made by Mr. H. K. Beale (representing Birmingham Children's Hospital), Mr. William Peach (representing the Stafford General Infirmary), Mr. Charles Perks (the Birmingham General Dispensary), Mr. Albert Cay (Warneford Hospital, Leamington), the Rev. H. V. Stuart (North Stafford Infirmary), Mr. R. Woodward (Kidderminster Infirmary), Mr. J. C. Vaudrey (Birmingham Ear and Throat Hospital), Mr. F. I. Hancock (West Bromwich Hospital), Mr. J. G. Dunn (Birmingham General Dispensary), Mr. Bache (West Bromwich Hospital).

Mr. William Peach suggested a verbal alteration in the preamble of the resolution, which was accepted by the Chairman.

The resolution was then put to the meeting and carried unanimously as follows: "That this meeting of voluntary hospitals of the Midlands, while welcoming any legislation which by reasonable arrangement will benefit the sick poor and continue to make use of the machinery of voluntary hospitals, is of opinion that the National Insurance Bill, if passed in its present form, will so gravely affect their income as to necessitate the curtailing of the amount and efficiency of their work, including a serious reduction in the number of beds they can support, and therefore urges that the Bill be so amended as to provide against such loss of income whilst preserving their independence and maintaining an alternative to the Poor Law infirmaries."

#### The Hospitals Represented.

Mr. F. S. Goodwin (Birmingham General Hospital) moved, Mr. T. Hilton (Kidderminster Infirmary) seconded, and it was unanimously resolved: "That a copy of the resolution, together with the statement submitted by Mr. Howard Collins, be sent to the public Press and to the members of Parliament for the districts represented and to each hospital of the district." On the proposal of Mr. Charles Perks, a cordial vote of thanks was passed to the Chairman.

The following is the list of those present:

Mr. Neville Chamberlain and Mr. F. S. Goodwin, Birmingham General Hospital.

Mr. W. A. Crosbee and Mr. W. Allport, Queen's Hospital.

Mr. C. C. Harding, Mr. E. H. Kannreuther, and Mr. H. K. Beale, Children's Hospital.
Mr. Mason, Eye Hospital.
Mr. S. C. Grew, Mr. W. J. Gell, and Mr. J. C. Vaudrey, Ear and Throat Hospital.
Mr. Howard Heaton, Mr. J. Keay, and Mr. George Bryson, Orthopædic Hospital.
Councillor Harris and Dr. Gilbert Smith, Skin Hospital.
Mr. Samuel Robinson, Homœopathic Hospital.
Mr. J. G. Dunn and Mr. Charles Perks, General Dispensary.
Mr. J. S. Neil, Wolverhampton General Hospital.
Mr. J. A. Rudd, Coventry and Warwick Hospital.
Mr. Albert Cay and Mr. Fred Smith, Warneford Hospital, Leamington.
Mr. G. W. Walton, Rugby Hospital.
Dr. Messiter and Mr. Joseph Nicholls, Guest Hospital, Dudley.
Mr. F. J. Hancock, Mr. Thomas F. Bache, and Mr. Thomas Jones, West Bromwich Hospital.
Mr. Harold Wigg and Rev. S. A. Millard, Walsall Hospital.
Mr. G. W. Evered and Miss J. E. Trindet, Stratford-on-Avon Hospital.
Mr. William Peach and Dr. F. M. Blumer, Staffordshire General Infirmary.
Rev. H. V. Stuart and Mr. C. E. Bullock, North Staffordshire Infirmary.
Mr. T. Hilton and Mr. R. Woodward, Kidderminster Infirmary.
Dr. H. Michie and Mr. C. N. Bass, Nottingham Samaritan Hospital for Women.
Mr. E. Forster, Derbyshire Royal Infirmary.
Mr. J. W. Taylor and Mr. W. Stevenson, Devonport Hospital, Buxton.
Mr. W. H. Head, Cheltenham General Hospital.
Mr. W. S. Tunbridge, Smallwood Hospital, Redditch.

